# Knowledge, attitudes, and practices of hemorrhoid patients regarding surgical treatment

**DOI:** 10.1038/s41598-026-59834-8

**Published:** 2026-07-10

**Authors:** Yan Hu, Xihong Wang, Lihong Chen

**Affiliations:** Department of Urology/Pelvic Floor Andrology, West China Fourth Hospital, Sichuan University, Chengdu, China

**Keywords:** Hemorrhoids, Health knowledge, attitudes, practice, Cross-sectional studies, Surgical procedures, operative, Patient education, Diseases, Medical research

## Abstract

This study aimed to assess the knowledge, attitudes, and practices (KAP) of patients with hemorrhoids concerning surgical treatment. This cross-sectional study was conducted between October 2023 and February 2025 in Chengdu, Sichuan Province, China and included patients diagnosed with hemorrhoids. Data were collected through a self-designed structured questionnaire, which included sections on sociodemographic characteristics and evaluated participants’ KAP related to surgical treatment. A total of 431 patients diagnosed with hemorrhoids participated in the study. The mean knowledge, attitude, and practice scores were 8.01 ± 3.07 (possible range: 0–18), 30.61 ± 2.16 (possible range: 7–35), and 34.68 ± 4.73 (possible range: 8–40), respectively. The results of the SEM analysis shown that attitude had a direct effect on practice (β = 0.378, *P* < 0.001). However, neither the direct effect of knowledge on attitude and practice nor the indirect effect of knowledge on practice was significant. Patients with hemorrhoids exhibited limited knowledge but generally positive attitudes and proactive practices regarding surgical treatment, with attitudes emerging as the primary driver of practice behaviors. These findings suggest that interventions aimed at improving patient outcomes should prioritize attitude-based education strategies, while also addressing knowledge gaps to support informed decision-making.

**Supplementary Information:**

The online version contains supplementary material available at 10.1038/s41598-026-59834-8.

## Introduction

Hemorrhoidal disease (HD) is among the most common anorectal disorders globally, with prevalence rates estimated at 4.4–36% of the general population in Western countries, affecting over 50% of individuals aged 50 years and above^1^. Studies from developed nations report that approximately 10 million people annually seek medical attention for hemorrhoids, resulting in substantial healthcare expenditure^2^. In China, recent epidemiological studies have revealed striking prevalence rates, with standardized detection rates of 17.7% in males and 43.7% in females across various age groups, demonstrating substantial gender disparities and regional variations throughout the country^3^. Additionally, a comprehensive survey by the Chinese Association of Chinese Medicine’s Anorectal Branch reported that anorectal diseases affect approximately 50.10% of urban and rural residents aged 18 and above in China, with hemorrhoids being the most common anorectal condition at 49.14%^4^. This condition significantly impacts patients’ quality of life by causing physical discomfort, psychological distress, and social limitations. The management of hemorrhoids encompasses a spectrum of approaches ranging from conservative methods and medical therapies to procedural interventions. Surgical treatment serves as a crucial and definitive option for advanced (grade III-IV) or refractory cases, offering significant advantages in terms of long-term symptom resolution and reduced recurrence rates compared to non-surgical alternatives. According to recent clinical studies, properly selected surgical approaches can provide complete symptom relief in up to 95% of cases, highlighting the importance of surgical interventions in the comprehensive management algorithm for hemorrhoidal disease^5^.

The comprehensive care pathway for hemorrhoid patients involves multiple critical phases, including not only surgical decision-making but also perioperative management and postoperative rehabilitation. Recent research has identified that approximately 70% of patients prefer minimally invasive surgical techniques when financially feasible, with postoperative complications constituting their primary concern^6^. Beyond surgical selection, postoperative rehabilitation—including wound care, pain management, and lifestyle modifications—significantly impacts treatment success and patient satisfaction. Studies demonstrate that structured rehabilitation protocols can reduce recovery time by up to 40% and decrease complication rates. Moreover, significant differences in awareness and preferences exist between patients with or without medical backgrounds, indicating that patients with medical backgrounds tend to have a clearer understanding of surgical procedures, while those without such training may rely more on physician explanations or informal advice, leading to varied levels of understanding. Socioeconomic status and psychological factors such as depression have been associated with lower scores in shared decision-making experiences and poorer adherence to postoperative care instructions, highlighting the complex interplay between patient characteristics and comprehensive surgical management^7^.

The Knowledge, Attitude, and Practice (KAP) framework provides a valuable structure for understanding patient behavior in healthcare contexts. This model posits that individuals’ healthcare decisions and behaviors are significantly influenced by their knowledge level, beliefs, and attitudinal dispositions toward specific interventions^8^. For hemorrhoid patients considering surgical treatment, KAP assessment can reveal critical insights into information gaps, misconceptions, and behavioral patterns that may affect treatment outcomes and satisfaction. Previous KAP studies have provided valuable insights into patient perspectives and behaviors in gastroenterology and surgery. In China, KAP investigations in surgical domains such as laparoscopic cholecystectomy have shown notable knowledge deficits regarding specific procedural details, despite generally favorable attitudes toward minimally invasive approaches^9^. KAP studies in anorectal disorders have examined patient perspectives on non-surgical management and prevention practices^10^, but comprehensive KAP studies specifically examining Chinese hemorrhoid patients’ perspectives on surgical interventions remain limited, creating a notable research gap in this high-prevalence region.

This study aims to investigate the KAP of hemorrhoid patients regarding surgical treatment in China, addressing the current research void. By identifying knowledge deficits, exploring attitudinal barriers, and examining decision-making practices, this research seeks to provide foundational data to improve patient education, enhance shared decision-making processes, and ultimately optimize surgical outcomes for hemorrhoid patients.

## Methods

### Study design and participants

This cross-sectional survey was conducted in Chengdu, Sichuan Province, between October, 2023, and February, 2025, and enrolled individuals diagnosed with hemorrhoids. Ethical approval for the study was obtained from the Clinical Research Management Committee of the Fourth Hospital of West China, Sichuan University (Approval number: HXSY-EC-2023101). All participants provided written informed consent prior to data collection.

The inclusion criteria were: (1) aged 18 years or older with a clinical diagnosis of hemorrhoids; (2) fully conscious and capable of independently completing the questionnaire; and (3) willing to participate voluntarily, with informed consent duly signed. The exclusion criteria were: (1) serious comorbid medical conditions or (2) history of psychiatric disorders or cognitive impairments that could interfere with their ability to understand or complete the survey.

### Questionnaire

The questionnaire was initially designed according to *Chinese Hemorrhoid Diagnosis and Treatment Guidelines (2020)* and *Consensus of experts on daytime operation for hemorrhoid diagnosis and treatment (2020 Edition).* After *the* initial design, a pilot survey involving 35 participants was conducted to assess the preliminary reliability of the questionnaire. After the pilot study, 8 questions in knowledge and attitude sections were removed because specialists found them of low relevance or patients found them unclear or unacceptable. Following these modifications, the Cronbach’s alpha coefficients for the knowledge, attitude, and practice dimensions were 0.669, 0.707, and 0.841, respectively, with an overall reliability coefficient of 0.811. All subsequent descriptions refer to the finalized version of the questionnaire. Three specialists in anorectal surgery and health education reviewed the revised questionnaire to assess the relevance and clarity of each item, which ensured preliminary content validity. Feedback from pilot participants confirmed that the questions in the revised questionnaire were understandable and acceptable to patients, supporting face validity. In the formal survey, the Cronbach’s alpha coefficients for the knowledge, attitude, and practice sections were 0.7719, 0.7975, and 0.9074, respectively, and the overall reliability coefficient was 0.8871.

The final questionnaire, developed in Chinese, consisted of four sections. The demographic section included 19 items assessing participants’ background characteristics. The knowledge section comprised 9 multiple-choice items, while the attitude and practice sections contained 7 and 8 items, respectively, both using five-point Likert-type scales. In the scoring system, responses in the knowledge section were evaluated based on accuracy: correct answers were awarded 2 points, “not sure” responses 1 point, and incorrect answers 0 points, resulting in a total possible score ranging from 0 to 18. The attitude section employed a five-point Likert scale ranging from “strongly agree” (5 points) to “strongly disagree” (1 point), while the item 6 was reverse-coded due to its negatively description, yielding a total score between 7 and 35. The practice section included 8 items, all using a five-point Likert scale ranging from “strongly agree” (5 points) to “strongly disagree” (1 point), with a total possible score ranging from 8 to 40. Attaining scores above 70% of the maximum in each section indicated adequate knowledge, positive attitude, and proactive practice^11^.

The questionnaire was administered electronically through Wenjuanxing, an online survey platform that generated a QR code linked to the survey. This QR code was provided to eligible patients during hospitalization, either as a printed copy or as a mobile screenshot. Upon admission, patients diagnosed with hemorrhoids were invited by the attending nurse to scan the QR code using WeChat and complete the questionnaire independently on their mobile devices. To ensure data integrity and prevent duplicate responses, each IP address was restricted to a single submission, and all items were set as mandatory. Completed questionnaires were reviewed by the research team for completeness, internal consistency, and logical coherence prior to inclusion in the final dataset.

### Statistical methods

Data were analyzed using R version 4.3.2 and Stata version 18.0. Knowledge, attitude, and practice scores were summarized using means and standard deviations (SD) for normally distributed data, or medians and interquartile ranges for non-normal distributions. Categorical variables were presented as frequencies and percentages. For comparing scores between groups, we used t-tests or Wilcoxon-Mann-Whitney tests for two-group comparisons, and ANOVA or Kruskal-Wallis tests for multiple groups, depending on data distribution. Relationships between knowledge, attitude, and practice scores were assessed using Pearson’s or Spearman’s correlation coefficients.

Factors associated with higher scores in each dimension were identified through logistic regression, with the median value of each dimension score used as the cutoff. Variables with *P* < 0.05 in univariate analyses were included in multivariate models. Finally, structural equation modeling (SEM) was used to explore relationships between knowledge, attitude, and practice. Model fit was evaluated using RMSEA, SRMR, TLI, and CFI, with acceptable thresholds of < 0.08 for RMSEA and SRMR, and > 0.80 for TLI and CFI.

## Results

### Basic information on the population

Among the 431 haemorrhoidal patients who participated in this study, 224 (52.0%) were aged 35–54 years, 249 (57.8%) had a BMI in the normal range, 346 (80.3%) lived in urban areas, 173 (40.1%) had a Bachelor’s Degree, 297 (68.9%) wiped with a dry tissue after bowel, 168 (39.0%) had been diagnosed for 2–5 years, 355 (82.4%) had mixed haemorrhoids, 164 (38.1%) had not been treated yet. The mean ± SD knowledge, attitude, and practice scores were 8.01 ± 3.07 (possible range: 0–18), 30.61 ± 2.16 (possible range: 7–35), and 34.68 ± 4.73 (possible range: 8–40), respectively. Participants’ knowledge scores were more likely to vary depending on age (*P* = 0.013), BMI (*P* = 0.027), residence (*P* = 0.011), education (*P* < 0.001), occupation type (*P* < 0.001), clean way after bowel (*P* = 0.016), and current treatment status (*P* = 0.003). Meanwhile, their practice scores were more likely to vary depending on occupation type (*P* = 0.010) and habit of eating spicy food (*P* = 0.003) (Table 1).

**Table 1:** Baseline characteristics.

Characteristics	*N*(%)	Knowledge	*P*	Attitude	*P*	Practice	*P*
		Mean (SD)		Mean (SD)		Mean (SD)	
Total score	431 (100.0)	8.01 (3.07)		30.61 (2.16)		34.68 (4.73)	
Age, years			**0.013**		0.761		0.586
18–34	155(36.0)	8.50 (2.90)		30.72 (2.31)		34.42 (4.89)	
35–54	224(52.0)	7.89 (3.09)		30.56 (2.13)		34.77 (4.42)	
55 and more	52(12.1)	7.06 (3.21)		30.46 (1.85)		35.06 (5.52)	
BMI, kg/m^2^			**0.027**		0.935		0.524
< 18.5	23(5.3)	9.13 (2.83)		30.39 (1.62)		35.74 (3.68)	
18.5–24.0	249(57.8)	8.20 (3.00)		30.58 (2.28)		34.67 (4.71)	
24.0–27.9	128(29.7)	7.70 (3.23)		30.63 (2.10)		34.80 (4.79)	
≥ 28.0	31(7.2)	6.94 (2.76)		30.87 (1.84)		33.48 (5.27)	
Residence			**0.011**		0.249		0.607
Rural	56(13.0)	6.98 (3.20)		30.59 (1.91)		34.96 (4.58)	
Urban	346(80.3)	8.22 (2.97)		30.55 (2.19)		34.58 (4.77)	
Suburban	29(6.7)	7.45 (3.54)		31.31 (2.21)		35.38 (4.59)	
Education			**< 0.001**		0.564		0.340
Junior high school or below	68(15.8)	6.54 (3.18)		30.43 (1.96)		34.62 (4.87)	
High school/vocational school	50(11.6)	7.36 (3.01)		30.78 (2.41)		34.84 (4.33)	
Associate degree	90(20.9)	7.84 (3.04)		30.86 (1.77)		35.62 (4.06)	
Bachelor’s degree	173(40.1)	8.52 (2.87)		30.51 (2.05)		34.20 (5.06)	
Master’s degree or above	50(11.6)	9.16 (2.87)		30.56 (3.04)		34.58 (4.80)	
Occupation			**< 0.001**		0.121		**0.010**
Non-healthcare-related profession	292(67.7)	7.78 (3.07)		30.74 (2.02)		34.90 (4.53)	
Healthcare-related profession	49(11.4)	10.39 (1.68)		30.67 (2.45)		35.94 (4.03)	
Retired	41(9.5)	7.59 (3.19)		30.32 (2.21)		34.12 (5.28)	
Unemployed	49(11.4)	7.33 (3.04)		29.98 (2.55)		32.55 (5.47)	
Monthly income, CNY			0.101		0.193		0.864
< 2000	28(6.5)	7.25 (3.65)		30.00 (2.71)		33.89 (4.84)	
2000–5000	103(23.9)	7.87 (3.06)		30.77 (1.90)		34.62 (4.23)	
5000–10,000	141(32.7)	7.78 (3.26)		30.68 (1.76)		34.82 (4.77)	
10,000–20,000	92(21.3)	7.99 (2.97)		30.22 (2.40)		34.42 (5.40)	
≥ 20,000	67(15.5)	9.03 (2.27)		31.00 (2.59)		35.16 (4.42)	
Smoking			0.852		0.054		0.284
Never smoked	327(75.9)	8.03 (3.14)		30.58 (2.12)		34.83 (4.75)	
Used to smoke	46(10.7)	7.80 (3.02)		30.13 (2.48)		33.83 (4.47)	
Still smoking	58(13.5)	8.03 (2.69)		31.12 (2.04)		34.50 (4.84)	
Habit of eating spicy food			0.195		0.500		**0.003**
Never eat spicy food	30(7.0)	7.10 (3.36)		30.87 (1.96)		35.73 (4.49)	
Eat mildly spicy food	297(68.9)	8.05 (3.00)		30.54 (2.25)		34.21 (4.81)	
Prefer very spicy food	104(24.1)	8.15 (3.16)		30.74 (1.97)		35.72 (4.39)	
Clean way after bowel			**0.016**		0.667		0.616
Wet wipes	84(19.5)	8.75 (3.20)		30.75 (2.05)		35.10 (4.22)	
Dry tissue	297(68.9)	7.77 (3.02)		30.60 (2.22)		34.63 (4.86)	
Water rinse or other methods	50(11.6)	8.18 (2.97)		30.40 (2.03)		34.28 (4.79)	
Years of hemorrhoids			0.539		0.478		0.184
Within 1 year	100(23.2)	8.28 (2.99)		30.29 (2.50)		33.89 (5.14)	
2–5 years	168(39.0)	7.78 (3.07)		30.76 (1.96)		35.23 (4.33)	
6–10 years	92(21.3)	8.05 (3.25)		30.64 (2.25)		34.33 (5.10)	
More than 10 years	71(16.5)	8.10 (2.93)		30.65 (1.96)		34.94 (4.41)	
Type of hemorrhoids			0.324		0.675		0.666
Internal hemorrhoids	45(10.4)	8.16 (3.39)		30.73 (2.58)		34.51 (4.71)	
External hemorrhoids	31(7.2)	8.65 (2.83)		30.48 (2.45)		34.23 (3.79)	
Mixed hemorrhoids	355(82.4)	7.93 (3.04)		30.60 (2.08)		34.74 (4.81)	
Previous treatment			0.196		0.999		0.940
No treatment	164(38.1)	7.69 (3.16)		30.68 (2.08)		34.71 (4.80)	
Conservative treatment	194(45.0)	8.16 (2.92)		30.59 (2.07)		34.68 (4.40)	
Surgical treatment	73(16.9)	8.30 (3.23)		30.51 (2.57)		34.60 (5.44)	
Current treatment			**0.003**		0.126		0.221
Conservative treatment	58(13.5)	8.95 (3.20)		30.16 (2.80)		34.02 (4.44)	
Surgical treatment	373(86.5)	7.86 (3.02)		30.68 (2.04)		34.78 (4.77)	

### Knowledge, attitude, and practice

The distribution of knowledge dimensions showed that the three questions with the highest number of participants choosing the ‘Not sure’ option were ‘Weakening of the anal cushion and supporting tissues, as well as spasms of the internal anal sphincter, are the primary causes of hemorrhoids.’ (K2) with 63.81%, and ‘Surgical treatment options for hemorrhoids include hemorrhoidectomy, stapled hemorrhoidopexy, and transanal hemorrhoidal dearterialization’ (K6) with 42.69% (Table S1).

Responses to the attitude dimension showed that 16.01% strongly agreed and 30.63% agreed that they worried about the recovery process after hemorrhoid surgery (A5). Meanwhile, 0.93% strongly disagreed and 6.03% disagreed that surgery is the best treatment option for hemorrhoids (A1), 1.86% strongly disagreed and 0.7% disagreed that surgery is very effective in relieving hemorrhoid pain (A3) (Table S2).

Responses to the practice dimension showed that 2.55% disagreed and 1.16% strongly disagreed that they would consult other patients about their experiences and advice regarding the procedure before surgery (P7), 2.09% disagreed and 0.7% strongly disagreed that they would proactively seek information and materials related to hemorrhoid surgery (P5), and 4.18% disagreed and 0.46% strongly disagreed that they would seek medical examination for hemorrhoids when experiencing rectal bleeding (P2) (Table S3).

### Univariate and multivariate analysis of knowledge, attitude, and practice dimensions

The median of the knowledge, attitude, and practice scores were used as the cut-off value for each dimension to divided the groups. Multivariate logistic regression showed that with Bachelor’s degree (OR = 2.747, 95% CI: [1.272,5.933], *P* = 0.010), with Master’s degree or above (OR = 5.491, 95% CI: [2.085,14.466], *P* = 0.001), being healthcare-related profession (OR = 7.740, 95% CI: [2.670,22.439], *P* < 0.001), with surgical treatment (OR = 2.267, 95% CI: [1.218,4.218)], *P* = 0.010) were independently associated with good knowledge (Table 2). Concurrently, with external hemorrhoids (OR = 2.811, 95% CI: [1.076,7.343], *P* = 0.035) was independently associated with positive attitude (Table 3). Moreover, attitude (OR = 1.397, 95% CI: [1.251,1.560], *P* < 0.001) was independently associated with proactive practice (Table 4).

**Table 2:** Univariate and multivariate analysis for knowledge dimension.

Knowledge	Univariate analysis		Multivariate analysis	
	OR (95%CI)	*P*	OR (95%CI)	*P*
Age, years				
18–34				
35–54	0.655 (0.432,0.989)	0.045	0.889 (0.540,1.462)	0.643
55 and more	0.440 (0.228,0.831)	0.012	0.724 (0.307,1.704)	0.459
BMI, kg/m^2^				
< 18.5				
18.5–24.0	0.405 (0.142,1.010)	0.066	0.489 (0.163,1.466)	0.201
24.0–27.9	0.302 (0.103,0.778)	0.018	0.458 (0.147,1.425)	0.177
≥ 28.0	0.168 (0.048,0.532)	0.004	0.353 (0.094,1.325)	0.123
Residence				
Rural				
Urban	2.512 (1.398,4.666)	0.003	0.998 (0.473,2.105)	0.996
Suburban	1.715 (0.679,4.340)	0.251	0.862 (0.296,2.514)	0.786
Education				
Junior high school or below				
High school/vocational school	1.852 (0.849,4.080)	0.122	1.523 (0.637,3.638)	0.344
Associate degree	2.541 (1.304,5.096)	0.007	1.570 (0.717,3.438)	0.260
Bachelor’s degree	3.991 (2.185,7.560)	< 0.001	2.747 (1.272,5.933)	0.010
Master’s degree or above	7.143 (3.220,16.686)	< 0.001	5.491 (2.085,14.466)	0.001
Occupation				
Non-healthcare-related profession				
Healthcare-related profession	9.686 (4.085,28.590)	< 0.001	7.740 (2.670,22.439)	< 0.001
Retired	0.951 (0.490,1.831)	0.880	1.786 (0.764,4.176)	0.181
Unemployed	0.585 (0.305,1.087)	0.096	0.747 (0.361,1.543)	0.430
Monthly income per capita, CNY				
< 2000				
2000–5000	1.223 (0.529,2.858)	0.637		
5000–10,000	1.015 (0.450,2.317)	0.971		
10,000–20,000	1.058 (0.453,2.497)	0.897		
≥ 20,000	2.067 (0.847,5.129)	0.112		
Smoking				
Never smoked				
Used to smoke	0.878 (0.471,1.631)	0.680		
Still smoking	1.027 (0.587,1.801)	0.927		
Habit of eating spicy food				
Never eat spicy food				
Eat mildly spicy food	1.763 (0.823,3.951)	0.153	1.615 (0.675,3.861)	0.281
Prefer very spicy food	2.178 (0.956,5.168)	0.069	1.644 (0.640,4.226)	0.302
Clean way after bowel				
Wet wipes				
Dry tissue	0.571 (0.346,0.934)	0.027	0.593 (0.344,1.024)	0.061
Water rinse or other methods	0.568 (0.278,1.151)	0.118	0.638 (0.297,1.369)	0.249
Years of hemorrhoids				
Within 1 year				
2–5 years	0.826 (0.502,1.355)	0.449		
6–10 years	1.103 (0.625,1.951)	0.735		
More than 10 years	0.815 (0.442,1.498)	0.510		
Type of hemorrhoids				
Internal hemorrhoids				
External hemorrhoids	1.212 (0.482,3.085)	0.684		
Mixed hemorrhoids	0.870 (0.464,1.620)	0.661		
Previous treatment				
No treatment				
Conservative treatment	1.471 (0.969,2.238)	0.070	1.579 (0.988,2.525)	0.056
Surgical treatment	1.597 (0.919,2.797)	0.099	2.267 (1.218,4.218)	0.010
Current treatment				
Conservative treatment				
Surgical treatment	0.496 (0.274,0.875)	0.018	0.936 (0.461,1.900)	0.855

**Table 3:** Univariate and multivariate analysis for attitude dimension.

Attitude	Univariate analysis		Multivariate analysis	
	OR (95%CI)	*P*	OR (95%CI)	*P*
Knowledge	0.987 (0.927,1.050)	0.668	0.981 (0.919,1.047)	0.563
Age, years				
18–34				
35–54	0.975 (0.646,1.471)	0.905		
55 and more	0.986 (0.525,1.861)	0.965		
BMI, kg/m^2^				
< 18.5				
18.5–24.0	0.956 (0.394,2.256)	0.919		
24.0–27.9	0.769 (0.308,1.875)	0.565		
≥ 28.0	0.934 (0.311,2.774)	0.902		
Residence				
Rural				
Urban	0.973 (0.550,1.713)	0.925		
Suburban	1.647 (0.660,4.280)	0.292		
Education				
Junior high school or below				
High school/vocational school	1.333 (0.638,2.815)	0.446		
Associate degree	1.111 (0.590,2.094)	0.744		
Bachelor’s degree	0.899 (0.511,1.577)	0.711		
Master’s degree or above	1.228 (0.589,2.580)	0.585		
Occupation				
Non-healthcare-related profession				
Healthcare-related profession	0.870 (0.474,1.605)	0.653	0.929 (0.488,1.770)	0.822
Retired	0.733 (0.379,1.414)	0.353	0.783 (0.403,1.524)	0.472
Unemployed	0.577 (0.310,1.060)	0.078	0.615 (0.330,1.145)	0.125
Monthly income, CNY				
< 2000				
2000–5000	1.652 (0.714,3.908)	0.243		
5000–10,000	1.699 (0.753,3.926)	0.205		
10,000–20,000	1.333 (0.571,3.181)	0.509		
≥ 20,000	1.747 (0.721,4.331)	0.220		
Smoking				
Never smoked				
Used to smoke	0.538 (0.282,1.004)	0.054	0.575 (0.302,1.095)	0.092
Still smoking	1.370 (0.778,2.460)	0.282	1.326 (0.743,2.368)	0.340
Habit of eating spicy food				
Never eat spicy food				
Eat mildly spicy food	0.591 (0.264,1.265)	0.184		
Prefer very spicy food	0.890 (0.375,2.037)	0.785		
Clean way after bowel				
Wet wipes				
Dry tissue	0.932 (0.570,1.515)	0.777		
Water rinse or other methods	0.727 (0.358,1.466)	0.373		
Years of hemorrhoids				
Within 1 year				
2–5 years	1.322 (0.805,2.176)	0.270		
6–10 years	1.041 (0.590,1.836)	0.890		
More than 10 years	1.598 (0.866,2.980)	0.136		
Type of hemorrhoids				
Internal hemorrhoids				
External hemorrhoids	2.995 (1.176,7.964)	0.024	2.811 (1.076,7.343)	0.035
Mixed hemorrhoids	2.007 (1.071,3.866)	0.032	1.878 (0.981,3.595)	0.057
Previous treatment				
No treatment				
Conservative treatment	1.333 (0.878,2.028)	0.178		
Surgical treatment	1.003 (0.577,1.744)	0.992		
Current treatment				
Conservative treatment				
Surgical treatment	1.293 (0.743,2.259)	0.363		

**Table 4:** Univariate and multivariate analysis for practice dimension.

Practice	Univariate analysis		Multivariate analysis	
	OR (95%CI)	*P*	OR (95%CI)	*P*
Knowledge	0.994 (0.935,1.058)	0.860	0.991 (0.923,1.064)	0.806
Attitude	1.409 (1.264,1.570)	< 0.001	1.397 (1.251,1.560)	< 0.001
Age, years				
18–34				
35–54	2.894 (0.727,11.518)	0.772		
55 and more	3.990 (0.944,16.862)	0.318		
BMI, kg/m^2^				
< 18.5				
18.5–24.0	2.065 (1.164,3.663)	0.471		
24.0–27.9	2.173 (1.199,3.938)	0.583		
≥ 28.0	1.698 (1.211,2.380)	0.255		
Residence				
Rural				
Urban	2.255 (0.922,5.515)	0.477		
Suburban	3.412 (1.297,8.979)	0.662		
Education				
Junior high school or below				
High school/vocational school	2.526 (1.058,6.033)	0.839		
Associate degree	3.098 (0.961,9.988)	0.705		
Bachelor’s degree	2.144 (0.920,4.997)	0.346		
Master’s degree or above	2.526 (1.058,6.033)	0.839		
Occupation				
Non-healthcare-related profession				
Healthcare-related profession	2.751 (0.934,8.101)	0.969	0.848 (0.420,1.710)	0.644
Retired	1.926 (0.994,3.731)	0.207	0.711 (0.343,1.474)	0.359
Unemployed	1.497 (0.991,2.260)	0.005	0.524 (0.260,1.053)	0.069
Monthly income, CNY				
< 2000				
2000–5000	3.398 (1.205,9.584)	0.637		
5000–10,000	4.748 (1.229,18.344)	0.286		
10,000–20,000	3.170 (1.204,8.346)	0.741		
v≥ 20,000	4.150 (1.312,13.126)	0.435		
Smoking				
Never smoked				
Used to smoke	1.595 (0.992,2.566)	0.019	0.522 (0.256,1.062)	0.073
Still smoking	2.496 (0.896,6.950)	0.755	0.691 (0.378,1.265)	0.231
Habit of eating spicy food				
Never eat spicy food				
Eat mildly spicy food	1.489 (1.071,2.070)	0.026	0.448 (0.186,1.079)	0.073
Prefer very spicy food	2.105 (1.175,3.772)	0.509	0.834 (0.322,2.159)	0.709
Clean way after bowel				
Wet wipes				
Dry tissue	2.181 (0.859,5.535)	0.319		
Water rinse or other methods	2.168 (1.026,4.579)	0.475		
Years of hemorrhoids				
Within 1 year				
2–5 years	5.775 (0.715,46.662)	0.028	1.604 (0.927,2.777)	0.092
6–10 years	3.395 (0.873,13.203)	0.488	1.091 (0.582,2.046)	0.785
More than 10 years	4.841 (0.903,25.961)	0.145	1.381 (0.704,2.709)	0.348
Type of hemorrhoids				
Internal hemorrhoids				
External hemorrhoids	2.128 (1.190,3.805)	0.550		
Mixed hemorrhoids	3.576 (0.937,13.645)	0.444		
Previous treatment				
No treatment				
Conservative treatment	2.615 (0.756,9.049)	0.852		
Surgical treatment	3.317 (0.860,12.801)	0.522		
Current treatment				
Conservative treatment				
Surgical treatment	3.939 (0.841,18.443)	0.265		

### Correlation and interaction analysis

**Fig. 1:**
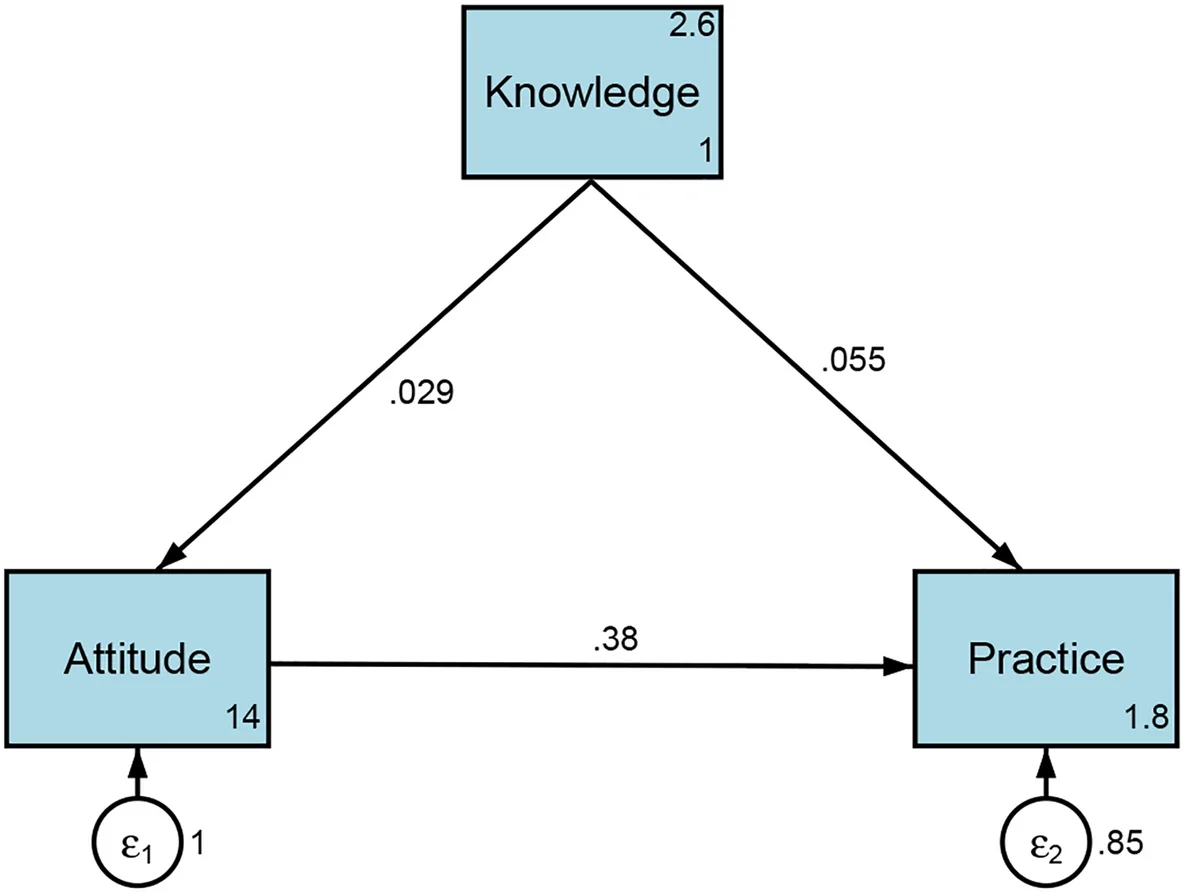
The structural equation modeling.

Further correlation analysis revealed attitude were positively correlated with practice (*r* = 0.404, *P* < 0.001). However, none of the correlations between knowledge with attitude and practice were significant (Table 5). The results of the SEM analysis shown that attitude had a direct effect on practice (β = 0.378, *P* < 0.001). However, neither the direct effect of knowledge on attitude and practice nor the indirect effect of knowledge on practice was significant (Table 6 and Fig. 1).

**Table 5:** Correlation analysis.

Spearman	Knowledge	Attitude	Practice
Knowledge	1.000		
Attitude	0.001 (*P* = 0.981)	1.000	
Practice	0.006 (*P* = 0.907)	0.404 (*P* < 0.001)	1.000

**Table 6:** SEM Results.

Model paths		Total effects		Direct effect		Indirect effect	
		β(95%CI)	*P*	β(95%CI)	*P*	β(95%CI)	*P*
Attitude							
	Knowledge	0.029 (−0.065, 0.123)	0.549	0.029 (−0.065, 0.123)	0.549		
Practice							
	Knowledge	0.065 (−0.028, 0.159)	0.172	0.055 (−0.033, 0.142)	0.220	0.011 (−0.025, 0.047)	0.550
	Attitude	0.378 (0.297, 0.459)	< 0.001	0.378 (0.297, 0.459)	< 0.001		

## Discussion

Patients with hemorrhoids demonstrated insufficient knowledge yet generally positive attitudes and proactive practices toward surgical treatment. Given that attitude was the primary determinant of practice, clinical interventions should focus on attitude-oriented education strategies to improve treatment behaviors, while simultaneously addressing knowledge gaps to ensure informed decision-making.

In this study, although most patients expressed favorable attitudes and reported active engagement in health-related practices, their overall knowledge about hemorrhoid surgery remained limited. This mismatch between attitudes, practices, and knowledge reflects a broader pattern observed in surgical care where patient behavior is not always driven by accurate understanding but rather shaped by symptom burden, trust in the healthcare system, and sociocultural norms^12,13^.

The relatively low level of knowledge observed in this study is consistent with previous research on chronic anorectal conditions, where even patients who have undergone treatment often remain unclear about the underlying causes of their disease, surgical indications, and postoperative care requirements^14,15^. Most participants showed awareness of lifestyle risk factors and symptoms but were uncertain about more technical aspects of hemorrhoid management, such as the specific type of surgery or the correct interpretation of postoperative recovery protocols. Similar findings have been reported in other surgical contexts, where patients’ comprehension of procedural details remains superficial unless targeted education is provided^16,17^. Interestingly, this knowledge gap was more pronounced among individuals with lower educational attainment and those not working in health-related fields, which reinforces the idea that formal education and professional exposure are strong enablers of surgical literacy. The difference in knowledge levels between medical and non-medical participants indicates that professional experience strongly influences understanding of surgical procedures. Therefore, practical education materials should be designed to help non-medical patients obtain accurate information and make informed choices.

Despite this limited knowledge, patients in our study generally held positive attitudes toward surgical treatment, with most expressing trust in the medical team and confidence in the effectiveness of surgery. This is consistent with earlier studies showing that patients tend to develop favorable attitudes toward surgical interventions when they believe these treatments offer definitive solutions to bothersome symptoms^18,19^.

However, concerns about surgical recovery and potential complications still emerged in the attitudinal responses. Although these concerns did not dominate the overall attitude scores, they are important because they may influence patients’ willingness to follow through with treatment when surgery is actually recommended. Previous studies have shown that preoperative anxiety and fear of pain are common barriers to surgical compliance, even when patients initially report willingness^20–22^. In light of this, clinicians should not assume that general positivity translates into action. Addressing these more nuanced fears through detailed counseling and real-life recovery stories from other patients could help mitigate last-minute refusals or noncompliance.

In terms of practices, participants reported a high level of engagement with their own care. Many stated that they actively sought information, monitored their lifestyle habits, followed medical advice, and maintained contact with healthcare providers. These patterns are encouraging and suggest a degree of personal responsibility and trust in the system. Importantly, both regression and SEM analyses indicated that these proactive behaviors were not driven by knowledge but were significantly associated with attitude. This finding echoes previous research suggesting that emotional readiness and belief in medical authority often serve as stronger motivators of health behavior than technical comprehension, especially in lower-resource or lower-literacy populations^23,24^. Therefore, interventions aiming to promote effective surgical decision-making should not rely solely on patient education materials. Instead, they should also incorporate techniques that reinforce patients’ confidence and perceived benefit of the treatment, such as motivational interviewing, visual testimonials, or group-based pre-surgical education.

While most patients demonstrated constructive practices, certain patterns in the data point to potential gaps. For example, individuals with less favorable socioeconomic conditions, such as those who were unemployed or had inconsistent dietary habits, tended to report slightly lower engagement, although these differences were not statistically significant in adjusted models. These trends mirror findings in broader health behavior literature, which suggests that structural constraints—such as job insecurity, low health literacy, and lack of social support—can undermine individuals’ ability to maintain consistent health practices^25,26^. Thus, identifying at-risk subgroups and providing them with tailored support—such as follow-up calls, digital reminders, or simplified education tools—may help close these behavior gaps.

The disconnect between knowledge and both attitude and practice in this study is particularly noteworthy. Although the KAP framework typically assumes that increased knowledge leads to more positive attitudes and, in turn, better practices, our findings suggest a different mechanism may be at play in surgical decision-making. This separation between knowledge and attitude contradicts traditional KAP assumptions but has been similarly observed in several other surgical populations, particularly when patients rely on doctors’ recommendations or peer experiences rather than formal understanding to form their opinions^14^. Similar findings were observed in patients with anorectal disorders in a multi-center study in Southern China, where positive attitudes toward surgical interventions were maintained regardless of knowledge levels, with 78.3% of participants indicating that physician recommendation was their primary decision factor^27^. This phenomenon may be attributed to the cultural context of healthcare in China, where patients often prioritize trust in healthcare providers over personal understanding of medical procedures, highlighting the importance of the physician-patient relationship in surgical decision-making^28^. Patients may act not because they understand all medical details but because they experience symptom-related distress, have strong trust in their doctors, or are influenced by peers who have undergone similar treatments. To translate these insights into actionable strategies, a multi-level approach is recommended. First, improve the clarity and accessibility of surgical information, particularly for patients with low education or no prior surgical experience. This may include using plain-language materials, infographics, and videos in outpatient settings. Second, invest in communication training for providers, helping them recognize attitudinal cues and respond with empathy and evidence-based reassurance^29,30^. Third, strengthen the continuity of care—through follow-ups, peer education, and multidisciplinary case discussions—so that patients remain supported throughout their surgical journey. Finally, health systems should integrate these efforts into broader quality improvement plans, ensuring that surgical care is not only technically sound but also emotionally acceptable and socially equitable^29^.

This study has several limitations that should be acknowledged. First, the cross-sectional design precludes the establishment of causal relationships between knowledge, attitudes, and practices. Second, the use of self-reported questionnaires may introduce recall bias or social desirability bias, potentially affecting the accuracy of the responses. Third, as the survey was conducted in a single geographic region, the generalizability of the findings to broader populations may be limited. Finally, although preliminary face and content validity were established, further psychometric validation such as factor analysis or Delphi method was not performed.

In conclusion, patients with hemorrhoids demonstrated insufficient knowledge but generally maintained positive attitudes and engaged in proactive practices regarding surgical treatment, with educational attainment and healthcare-related occupations significantly associated with higher knowledge levels. Targeted health education interventions, particularly for individuals with lower educational backgrounds and non-medical professions, are warranted to enhance informed decision-making and optimize treatment outcomes in hemorrhoidal care.

## Supplementary Information

Below is the link to the electronic supplementary material.

Supplementary Material 1

Supplementary Material 2

## Data Availability

All data generated or analysed during this study are included in this published article.
