# Supplementary material for: Knowledge, attitudes, and practices of hemorrhoid patients regarding surgical treatment
Source: Sci Rep. 2026 Jul 10;16:26520. doi: 10.1038/s41598-026-59834-8 (PMC13503939; doi:10.1038/s41598-026-59834-8)
Supplement: Supplementary file 1 [file 41598_2026_59834_MOESM1_ESM.docx]

Questionnaire ID

Dear Patient,

We are researchers from the Fourth Hospital of West China, Sichuan University, and we sincerely invite you to participate in our study. This research aims to understand hemorrhoid patients' knowledge, attitudes, and practices regarding surgical treatment, providing a basis for developing scientific intervention strategies. This may help more people improve their health in the future. Participation in this study is voluntary, and the study has been reviewed and approved by the Ethics Committee. If you agree to participate, please refer to the following instructions:

1.Please complete the questionnaire. There are no right or wrong answers; simply answer based on your actual situation. If you have any questions during the process, feel free to ask us. Once completed, please submit it promptly.

2.This study is a simple questionnaire survey that will not cause any harm to your physical or mental health. However, it involves some personal information, such as your gender and age. We will strictly protect your privacy and ensure that your information is not disclosed, so please feel assured when filling it out.

3.As a participant, you may access relevant study information and research progress at any time. If you decide to withdraw from the study, please inform us, and your data will not be included in the research results.

Finally, we sincerely appreciate your valuable time and support for our scientific research!

□I have read and agree to allow the collected data to be used for scientific research.

Informed Consent Signature:

Participation Date: ____Year ____Month ____Day

| **Part 1 Basic Information** | | |
| --- | --- | --- |
| **1. Your gender:** | a. Male | b. Female |
| **2. Your age: ____. (Numbers only; please set integer validation in the questionnaire system)** | | |
| **3. Your height: ____ cm. (Numbers only; please set validation in the questionnaire system)** | | |
| **4. Your weight: ____ kg. (Numbers only; please set validation in the questionnaire system)** | | |
| **5. Your place of residence:** | a. Rural  b. Urban  c. Suburban | |
| **6. Your education level:** | a. Junior high school or below b. High school/vocational school c. Associate degree d. Bachelor's degree e. Master's degree or above | |
| **7. Your occupation type:** | a. Healthcare-related profession b. Non-healthcare-related profession c. Retired d. Unemployed | |
| **8. Your average monthly household income per capita in the past year (including in-kind and rental income):** | a.<2000  b.2000-5000  c.5000-10000  d.10000-20000  e.>20000 | |
| **9. Your marital status:** | a. Single b. Married c. Divorced d. Widowed | |
| **10. Type of medical insurance:** | a. Only social medical insurance (e.g., employee medical insurance, New Rural Cooperative Medical Scheme, urban residents' insurance, etc.) b. Only commercial medical insurance c. Both social and commercial medical insurance d. No insurance | |
| **11. Do you have a smoking habit?** | a. Never smoked b. Used to smoke c. Still smoking | |
| **12.Do you have a drinking habit?** | a. Never drink b. Used to drink c. Still drink regularly | |
| **13. Do you have a habit of eating spicy food?** | a. Never eat spicy food b. Eat mildly spicy food c. Prefer very spicy food | |
| **14. In your daily life or work, how long do you stand or sit for prolonged periods?** | a. Less than 2 hours b. 2–4 hours c. More than 4 hours | |
| **15. Do you have a regular bowel movement habit? (Irregular habits include using a phone or reading while on the toilet.)** | a. Yes b. No | |
| **16. How do you clean yourself after a bowel movement?** | a. Dry tissue b. Wet wipes c. Water rinse d. Other methods | |
| **16. How many years have you been diagnosed with hemorrhoids? ____. (Numbers only; please set validation in the questionnaire system)** | | |
| **17. What type of hemorrhoids do you have?** | a. Internal hemorrhoids b. External hemorrhoids c. Mixed hemorrhoids | |
| **18. What was your previous treatment for hemorrhoids?** | a. No treatment b. Conservative treatment (diet adjustment, oral or topical medication) c. Surgical treatment | |
| **19. What is your current treatment for hemorrhoids?** | a. Conservative treatment (diet adjustment, oral or topical medication) b. Surgical treatment | |

| **Part 2 Knowledge of Hemorrhoids and Surgical Treatment** | | | |  |
| --- | --- | --- | --- | --- |
| **For the following statements, please indicate your level of understanding:** | | | | |
| **1. Weakening of the anal cushion and supporting tissues, as well as spasms of the internal anal sphincter, are the primary causes of hemorrhoids.** | a. True | b. False | c. Not sure | |
| **2. Unhealthy lifestyle habits (such as alcohol consumption, spicy food, prolonged standing or walking) and improper defecation habits increase the risk of hemorrhoids.** | a. True | b. False | c. Not sure | |
| **3. Common symptoms of hemorrhoids include bleeding, swelling, prolapse, pain, itching, and anal discomfort, which significantly affect the patient's quality of life.** | a. True | b. False | c. Not sure | |
| **4. Recurrent bleeding in hemorrhoid patients can lead to secondary anemia, and in some cases, severe bleeding may require emergency hospitalization and blood transfusion.** | a. True | b. False | c. Not sure | |
| **5. Patients with grade I–III hemorrhoids who have not achieved satisfactory results with conservative and/or instrumental treatments, or those with grade IV hemorrhoids willing to undergo surgery, may consider surgical treatment.** | a. True | b. False | c. Not sure | |
| **6 Surgical treatment options for hemorrhoids include hemorrhoidectomy, stapled hemorrhoidopexy (SH), and transanal hemorrhoidal dearterialization (THD).** | a. True | b. False | c. Not sure | |
| **7. In cases of acute hemorrhoidal emergencies, such as large thrombosis, severe pain, or excessive bleeding, surgical removal is recommended as soon as possible (within 72 hours).** | a. True | b. False | c. Not sure | |
| **8. Without proper dietary and lifestyle adjustments after hemorrhoid surgery, recurrence is likely.** | a. True | b. False | c. Not sure | |
| **9. Postoperative wound care for hemorrhoid surgery requires daily dressing changes.** | a. True | b. False | c. Not sure | |

| **Part 3 Attitudes and Perceptions Toward Hemorrhoid Surgery** | | | | | |
| --- | --- | --- | --- | --- | --- |
| **1. I believe that surgery is the best treatment option for hemorrhoids.** | a. strongly agree | b. agree | c. neutral | d. disagree | e. strongly disagree |
| **2. I have full confidence in the safety of hemorrhoid surgery.** | a. very confident | b. confident | c. uncertain | d. not confident | e. not confident at all |
| **3. I believe that surgery is very effective in relieving hemorrhoid pain.** | a. strongly agree | b. agree | c. neutral | d. disagree | e. strongly disagree |
| **4. If surgery is needed, I am willing to undergo hemorrhoid surgery.** | a. strongly agree | b. agree | c. neutral | d. disagree | e. strongly disagree |
| **5. I am worried about the recovery process after hemorrhoid surgery.** | a. strongly agree | b. agree | c. neutral | d. disagree | e. strongly disagree |
| **6. I believe hemorrhoid surgery will improve my overall health.** | a. strongly agree | b. agree | c. neutral | d. disagree | e. strongly disagree |
| **7. I trust the medical team performing hemorrhoid surgery.** | a. strongly agree | b. agree | c. neutral | d. disagree | e. strongly disagree |

| **Part 4 Behaviors and Practices Regarding Hemorrhoids and Surgical Treatment** | | | | | |
| --- | --- | --- | --- | --- | --- |
| **1. I actively learn about hemorrhoidal diseases.** | a. strongly agree | b. agree | c. neutral | d. disagree | e. strongly disagree |
| **2. If I experience rectal bleeding, I will seek medical examination for hemorrhoids.** | a. strongly agree | b. agree | c. neutral | d. disagree | e. strongly disagree |
| **3. I consciously manage my diet and lifestyle to prevent the worsening of hemorrhoids.** | a. strongly agree | b. agree | c. neutral | d. disagree | e. strongly disagree |
| **4. I follow the doctor's recommendations for treatment.** | a. strongly agree | b. agree | c. neutral | d. disagree | e. strongly disagree |
| **If surgery is needed:** |  |  |  |  |  |
| **5. I proactively seek information and materials related to hemorrhoid surgery.** | a. strongly agree | b. agree | c. neutral | d. disagree | e. strongly disagree |
| **6. I discuss the risks and possible complications of the surgery with my doctor.** | a. strongly agree | b. agree | c. neutral | d. disagree | e. strongly disagree |
| **7. Before surgery, I consult other patients about their experiences and advice regarding the procedure.** | a. strongly agree | b. agree | c. neutral | d. disagree | e. strongly disagree |
| **8. When receiving a follow-up call from the hospital, I proactively provide updates on my postoperative recovery.** | a. strongly agree | b. agree | c. neutral | d. disagree | e. strongly disagree |

**Thank you again for filling out our questionnaire, the information you provided will be valuable to us in the future!**

**Thank you for filling out our questionnaire！**

If you have any comments and suggestions on this survey, we would be honored to hear your voice.

Opinions and Suggestions: （optional）

In order to this questionnaire research can actually play a role in promoting the smooth development of the future return visit, if you are willing to leave your contact information, we would be grateful!

Your phone number：	（optional）
