# Supplementary material for: Knowledge, attitudes, and practices of hemorrhoid patients regarding surgical treatment
Source: Sci Rep. 2026 Jul 10;16:26520. doi: 10.1038/s41598-026-59834-8 (PMC13503939; doi:10.1038/s41598-026-59834-8)
Supplement: Supplementary file 2 [file 41598_2026_59834_MOESM2_ESM.docx]

**Supplementary table 1. Distribution of knowledge dimension responses**

| **Knowledge** | True | False | Not sure |
| --- | --- | --- | --- |
| 1. Weakening of the anal cushion and supporting tissues, as well as spasms of the internal anal sphincter, are the primary causes of hemorrhoids. | 136 (31.55%) | 20 (4.64%) | 275 (63.81%) |
| 2. Unhealthy lifestyle habits (such as alcohol consumption, spicy food, prolonged standing or walking) and improper defecation habits increase the risk of hemorrhoids. | 401 (93.04%) | 4 (0.93%) | 26 (6.03%) |
| 3. Common symptoms of hemorrhoids include bleeding, swelling, prolapse, pain, itching, and anal discomfort, which significantly affect the patient's quality of life. | 388 (90.02%) | 4 (0.93%) | 39 (9.05%) |
| 4. Recurrent bleeding in hemorrhoid patients can lead to secondary anemia, and in some cases, severe bleeding may require emergency hospitalization and blood transfusion. | 333 (77.26%) | 17 (3.94%) | 81 (18.79%) |
| 5. Patients with grade I–III hemorrhoids who have not achieved satisfactory results with conservative and/or instrumental treatments, or those with grade IV hemorrhoids willing to undergo surgery, may consider surgical treatment. | 298 (69.14%) | 3 (0.7%) | 130 (30.16%) |
| 6. Surgical treatment options for hemorrhoids include hemorrhoidectomy, stapled hemorrhoidopexy (SH), and transanal hemorrhoidal dearterialization (THD). | 243 (56.38%) | 4 (0.93%) | 184 (42.69%) |
| 7. In cases of acute hemorrhoidal emergencies, such as large thrombosis, severe pain, or excessive bleeding, surgical removal is recommended as soon as possible (within 72 hours). | 273 (63.34%) | 7 (1.62%) | 151 (35.03%) |
| 8. Without proper dietary and lifestyle adjustments after hemorrhoid surgery, recurrence is likely. | 397 (92.11%) | 8 (1.86%) | 26 (6.03%) |
| 9. Postoperative wound care for hemorrhoid surgery requires daily dressing changes. | 344 (79.81%) | 12 (2.78%) | 75 (17.4%) |

**Supplementary table 2. Distribution of attitude dimension responses**

| **Attitude** | Strongly agree | Agree | Neutral | Disagree | Strongly disagree |
| --- | --- | --- | --- | --- | --- |
| 1. I believe that surgery is the best treatment option for hemorrhoids. | 137 (31.79%) | 146 (33.87%) | 118 (27.38%) | 26 (6.03%) | 4 (0.93%) |
| 2. I have full confidence in the safety of hemorrhoid surgery. | 188 (43.62%) | 195 (45.24%) | 40 (9.28%) | 4 (0.93%) | 4 (0.93%) |
| 3. I believe that surgery is very effective in relieving hemorrhoid pain. | 148 (34.34%) | 208 (48.26%) | 64 (14.85%) | 8 (1.86%) | 3 (0.7%) |
| 4. If surgery is needed, I am willing to undergo hemorrhoid surgery. | 189 (43.85%) | 213 (49.42%) | 23 (5.34%) | 3 (0.7%) | 3 (0.7%) |
| 5. I am worried about the recovery process after hemorrhoid surgery. | 69 (16.01%) | 132 (30.63%) | 145 (33.64%) | 70 (16.24%) | 15 (3.48%) |
| 6. I believe hemorrhoid surgery will improve my overall health. | 185 (42.92%) | 211 (48.96%) | 26 (6.03%) | 6 (1.39%) | 3 (0.7%) |
| 7. I trust the medical team performing hemorrhoid surgery. | 241 (55.92%) | 168 (38.98%) | 15 (3.48%) | 4 (0.93%) | 3 (0.7%) |

**Supplementary table 3. Distribution of practice dimension responses**

| **Practice** | Strongly agree | Agree | Neutral | Disagree | Strongly disagree |
| --- | --- | --- | --- | --- | --- |
| 1. I actively learn about hemorrhoidal diseases. | 187 (43.39%) | 170 (39.44%) | 66 (15.31%) | 6 (1.39%) | 2 (0.46%) |
| 2. If I experience rectal bleeding, I will seek medical examination for hemorrhoids. | 178 (41.3%) | 170 (39.44%) | 63 (14.62%) | 18 (4.18%) | 2 (0.46%) |
| 3. I consciously manage my diet and lifestyle to prevent the worsening of hemorrhoids. | 206 (47.8%) | 169 (39.21%) | 47 (10.9%) | 8 (1.86%) | 1 (0.23%) |
| 4. I follow the doctor's recommendations for treatment. | 246 (57.08%) | 175 (40.6%) | 7 (1.62%) | 2 (0.46%) | 1 (0.23%) |
| 5. I proactively seek information and materials related to hemorrhoid surgery. | 209 (48.49%) | 169 (39.21%) | 41 (9.51%) | 9 (2.09%) | 3 (0.7%) |
| 6. I discuss the risks and possible complications of the surgery with my doctor. | 206 (47.8%) | 171 (39.68%) | 45 (10.44%) | 8 (1.86%) | 1 (0.23%) |
| 7. Before surgery, I consult other patients about their experiences and advice regarding the procedure. | 184 (42.69%) | 179 (41.53%) | 52 (12.06%) | 11 (2.55%) | 5 (1.16%) |
| 8. When receiving a follow-up call from the hospital, I proactively provide updates on my postoperative recovery. | 247 (57.31%) | 169 (39.21%) | 13 (3.02%) | 1 (0.23%) | 1 (0.23%) |
